# Stable Polymer-Lipid Hybrid Nanoparticles Based on *mcl*-Polyhydroxyalkanoate and Cationic Liposomes for mRNA Delivery

**DOI:** 10.3390/pharmaceutics16101305

**Published:** 2024-10-07

**Authors:** Sergey M. Shishlyannikov, Ilya N. Zubkov, Vera V. Vysochinskaya, Nina V. Gavrilova, Olga A. Dobrovolskaya, Ekaterina A. Elpaeva, Mikhail A. Maslov, Andrey Vasin

**Affiliations:** 1 Institute of Biomedical Systems and Biotechnology, Peter the Great Saint Petersburg Polytechnic University, 29 Politechnicheskaya St., 195251 Saint Petersburg, Russia; ilyagosldstein@gmail.com (I.N.Z.); veravv2509@gmail.com (V.V.V.); daughtervgater@gmail.com (N.V.G.); vasin_av@spbstu.ru (A.V.); 2 Smorodintsev Research Institute of Influenza, 15/17 Prof. Popova Street, 197022 Saint Petersburg, Russia; dobrovolskaya.od@gmail.com (O.A.D.); elpaevak@gmail.com (E.A.E.); 3 M.V. Lomonosov Institute of Fine Chemical Technologies, Rtu Mirea, 86 Vernadsky Ave., 119454 Moscow, Russia; mamaslov@mail.ru

**Keywords:** polymer–lipid nanoparticles, *mcl*-polyhydroxyalkanoate, cationic liposome, mRNA transfection, ionic strength

## Abstract

**Background/Objectives:** The development of polymer–lipid hybrid nanoparticles (PLNs) is a promising area of research, as it can help increase the stability of cationic lipid carriers. Hybrid PLNs are core–shell nanoparticle structures that combine the advantages of both polymer nanoparticles and liposomes, especially in terms of their physical stability and biocompatibility. Natural polymers such as polyhydroxyalkanoate (PHA) can be used as a matrix for the PLNs’ preparation. **Methods**: In this study, we first obtained stable cationic hybrid PLNs using a cationic liposome (CL) composed of a polycationic lipid 2X3 (1,26-bis(cholest-5-*en*-3β-yloxycarbonylamino)-7,11,16,20-tetraazahexacosane tetrahydrochloride), helper lipid DOPE (1,2-dioleoyl-*sn*-glycero-3-phosphoethanolamine), and the hydrophobic polymer *mcl*-PHA, which was produced by the soil bacterium *Pseudomonas helmantisensis* P1. **Results**: The new polymer-lipid carriers effectively encapsulated and delivered model mRNA-eGFP (enhanced green fluorescent protein mRNA) to BHK-21 cells. We then evaluated the role of *mcl*-PHA in increasing the stability of cationic PLNs in ionic solutions using dynamic light scattering data, electrophoretic mobility, and transmission electron microscopy techniques. **Conclusions**: The results showed that increasing the concentration of PBS (phosphate buffered saline) led to a decrease in the stability of the CLs. At high concentrations of PBS, the CLs aggregate. In contrast, the presence of isotonic PBS did not result in the aggregation of PLNs, and the particles remained stable for 120 h when stored at +4 °C. The obtained results show that PLNs hold promise for further in vivo studies on nucleic acid delivery.

## 1. Introduction

Currently, in addition to the development of new lipid carriers, hybrid polymer and polymer–lipid nanoparticles (PLNs) are actively being studied as promising mRNA carriers [1,2,3,4]. Cationic liposomes (CLs) are widely used as nucleic acid (NA) delivery systems, forming relatively stable nanoscale complexes with a high degree of mRNA encapsulation and providing protection against enzymatic degradation [5]. Despite the fact that lipid nanoparticles based mRNA vaccines against COVID-19 have been approved for clinical use by US and EU regulators [6,7], the development of efficient carriers based on CLs is still an important area of the research. However, there are still some limitations to use of these drug delivery systems. One significant challenge is the decrease in the stability of cationic lipoplexes with mRNA during storage [8]. Over time, the mRNA and ionized lipid components of CLs undergo hydrolytic and oxidative degradation, leading to instability under different storage conditions [9,10]. This instability encourages the development of more stable formulations. Another significant disadvantage of CLs is their high positive charge, which can lead to interactions with serum/plasma proteins that are more active than with neutral or negatively charged liposomes [11,12]. These interactions can cause side effects both in vitro and in vivo. PEGylation of liposomes “shields” the positive charge and reduces the negative effects of the interaction between cationic carriers and anionic molecules in vivo [13,14]. Despite the fact that PEGylated liposomes have been approved for clinical use, PEG can cause some side effects in humans [15].

Polymers play a crucial role in enhancing the stability and modifying physical-chemical characteristics of various hybrid PLNs [16]. Their incorporation into these materials can lead to significant improvements in performance across various applications [17]. The use of biodegradable and biocompatible polymers is a promising alternative to PEG for increasing the stability and maintaining the effectiveness for clinical applications as delivery vehicles [2,18,19,20]. For example, PLNs-based on the hydrophobic polymer polylactide (PLA) are being investigated as carriers for a wide range of pharmaceutical compounds [21], including mRNA [22]. To improve their physical properties, PLAs are usually blended with plasticizers (PEG, polypropylene glycol (PPG), etc.) that may cause some side effects [23]. A promising alternative to PLAs and other synthetic polymers is the biodegradable, elastic polymers medium-chain-length polyhydroxyalkanoates (*mcl*-PHAs). PHAs are naturally occurring, hydrophobic energy-storage materials that form granules within the bacterial cytoplasm [24], making them promising candidates for drug delivery applications. The biodegradation rate of PHAs is significantly higher than that of PLAs [23]. PHAs have excellent biocompatibility [25], and biotechnological production of PHAs allows for adjusting their structure and molecular weight, thereby extending their functionality, which is an important factor in the development of stable carriers [26]. Therefore, PLNs made up of PHA and amphiphilic lipids could be promising materials for clinical applications [27], as their unique composition ensures the absorption and controlled release of active substances over a specific period of time, as well as complete degradation of the carrier in the body [25,28].

We have previously shown that CLs based on the polycationic amphiphile 2X3 (1,26-bis(cholest-5-*en*-3β-yloxycarbonylamino)-7,11,16,20-tetraazahexacosane tetrahydrochloride) and helper lipid DOPE (1,2-dioleoyl-*sn*-glycero-3-phosphoethanolamine) efficiently deliver mRNAs into eukaryotic cells [29].

The aim of this work was to develop stable PLNs based on *mcl*-PHA, polycationic lipid 2X3, and helper lipid DOPE for in vitro delivery of mRNA. The synthesis was performed using a modified oil-in-water emulsion method with sonication. Intracellular delivery efficiency was evaluated using the model mRNA-eGFP (enhanced green fluorescent protein mRNA). In addition, the comparative study on the influence of ionic strength on the stability of PLNs and CLs was performed. The data were mainly obtained by size and zeta potential measurements, flow cytometry analysis, fluorescence microscopy, and transmission electron microscopy (TEM). These results provide valuable insights into the impact of *mcl*-PHA on the stability of cationic PLNs, which can be used to optimize the design and development of mRNA delivery systems for future applications.

## 2. Materials and Methods

### 2.1. Materials

DOPE was purchased from Avanti Polar Lipids (Birmingham, AL, USA). Dulbecco’s modified Eagle’s medium (DMEM), penicillin/streptomycin and Lipofectamine MessengerMAX (MM) were purchased from Invitrogen (Carlsbad, CA, USA). All other reagents were of analytical grade and supplied by Sigma Chemical Co. (St. Louis, MO, USA).

### 2.2. Preparation of mcl-PHA, Produced by the Soil Bacterium Pseudomonas Helmanticensis P1

The *mcl*-Poly-3-hydroxyalkanoate (*mcl*-PHA) sample used in this study was obtained using the SDS-resistant bacteria *Pseudomonas helmanticensis* P1 [30]. The culture medium contained 0.5 g/L NH_4_Cl, 0.5 g/L (NH_4_)_2_SO_4_, 3 g/L Na_2_HPO_4_∙12H_2_O, 2 g/L KH_2_PO_4_∙H_2_O, 0.01 g/L MgSO_4_, and 20 g/L mixture of sodium oleate and stearate. The acidity of the medium was adjusted to pH 7 using a 10% aqueous NaOH. The inoculum was prepared in 1 L Erlenmeyer flasks (150 mL of culture medium per flask) on an incubator shaker at 28 °C and 250 rpm for 48 h. The ratio between the volumes of inoculate and the medium for the main cultivation was 1:5. The main cultivation was carried out in 2 L Erlenmeyer flasks on an incubator shaker at 28 °C and 250 rpm for 48 h (300 mL of medium per flask). At the end of cultivation, the biomass of *P. helmanticensis* P1 was separated by centrifugation at 10,000 g. The resulting biomass was washed three times with isotonic PBS to remove substrate residues. The biomass was dried at 105 °C for 24 h.

A sample of dry biomass (2 g) was washed three times with 100 mL of 96% ethanol at 25 °C to remove membrane lipids and fatty acid derivatives (including triacylglycerides). After washing with ethanol, the biomass was dried again at 105 °C for 24 h. *mcl*-PHA was extracted from dry biomass using *n*-hexane. Ethanol-washed and dried biomass was placed in a flask, 100 mL of *n*-hexane was added, and stirred on a magnetic stirrer for 24 h at 25 °C. The biomass was separated by filtration through a paper filter and the extraction was repeated twice. The extracts were combined and evaporated. The resulting dry residue (650 mg) was dissolved in 30 mL of *n*-hexane. The solution was filtered twice through a column containing 15 mL (7 g) of activated charcoal to remove endotoxins and lipids. The eluate was filtered through a reduced cellulose filter (0.2 µm) to remove the charcoal particles. The filtered eluate was evaporated. The isolated polymer was dried at 100 °C for 24 h to obtain 420 mg of *mcl*-PHA.

The composition of monomers in the polymer structure and the sample purity were determined by GC-MS as described earlier [24]. Molecular weight and polydispersity of the *mcl*-PHA were determined by exclusion chromatography as described previously [31].

### 2.3. In Vitro Transcription

mRNA encoding green fluorescent protein (mRNA-eGFP) was obtained by in vitro transcription (IVT) using the commercial HighYield T7 ARCA mRNA Synthesis Kit (Jena Bioscience, Jena, Germany, #RNT-102). As a template for the IVT reaction, 1 µg of lyanerized plasmid DNA pJet_GFP containing the T-7 promoter and the target GFP gene was used. During the IVT reaction, modified nucleotides were used: pseudouridine-5’-triphosphate and 5-methylcytidine-5’-triphosphate, as well as the cap structure (ARCA) (Jena Bioscience, Jena, Germany#RNT-102), to increase the translation efficiency of the resulting mRNA. We selected the optimal ARCA/GTP ratio, which was 4:1 in the IVT reaction, which ensured the maximum yield of mRNA containing the cap structure.

Using the HighYield T7 Cy5 RNA Labeling Kit (Jena Bioscience, Jena, Germany, #RNT-102), we obtained mRNA-eGFP containing a modified nucleotide labeled with the fluorescent Cyanine5 (Cy5) label (mRNA-Cy5). UTP-X-CY5 (also known as Cy5-17-UTP) is efficiently inserted into mRNA during IVT as a replacement for its natural UTP counterpart. Replacing 35% of UTP with UTP-X-Cy5 leads to an optimal balance between the efficiency of the IVT reaction and the labeling efficiency of the resulting mRNA.

After the IVT reaction, the template plasmid DNA was removed by treating the sample with DNAse (Invitrogen, Waltham, MA, USA, #AM1344). The resulting capped mRNA-eGFP and mRNA-Cy5 were polyadenylated using a commercial Poly(A) Tailing Enzyme Test kit (Jena Bioscience, Jena, Germany, #RNT-004), followed by purification by precipitation with lithium chloride, according to the manufacturer’s protocol. Samples of the resulting mRNA containing the cap structure, polyA structure, and modified nitrogenous bases were analyzed by electrophoresis under denaturing conditions. A total of 500 ng of the mRNA was mixed with an equivalent volume of Gel Loading Buffer II (Invitrogen, Waltham, MA, USA, #AM1344) and incubated for 10 min at 80 °C. The samples were then added to a 1% agarose gel containing 0.5 µg/mL ethidium bromide and separated electrophoretically in a 1×MOPS buffer. The electrophoresis results were detected using a ChemiDoc device (Bio-Rad Laboratories, Hercules, CA, USA).

### 2.4. Preparation of the Cationic PLNs

*mcl*-PHA nanoparticles stabilized with cationic amphiphile 2X3 and the helper lipid DOPE were obtained using a modified oil-in-water emulsion method based on [31,32]. We prepared four types of PLNs, containing 2X3 (PHA-2X3) or 2X3 and DOPE with molar ratios 1:1 (PHA-2X3-DOPE 1:1), 1:2 (PHA-2X3-DOPE 1:2), or 1:3 (PHA-2X3-DOPE 1:3).

**Figure 1:**
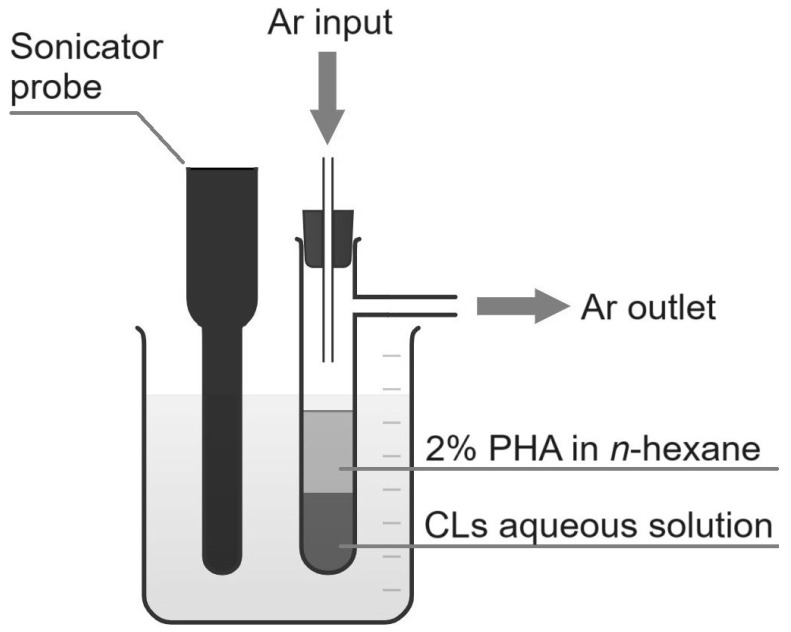
Reactor for the synthesis of PLNs. Reaction tube and the sonicator probe are placed in a beaker with water. During the synthesis and under sonication, the organic solvent (*n*-hexane) is removed by evaporation in argon stream.

To obtain lipid solutions, 1 mg/mL stock solutions of 2X3 or DOPE in CHCl_3_/MeOH (4:1 vol.) were mixed in the amounts indicated in Table 1. After that, the lipids mixtures (50 µL of each; total lipid concentration of 1 mg/mL) were dried using an argon stream. A total of 50 µL of nuclease-free water was added and the mixture was sonicated for 5 min (USDN-2T, «Academpribor»; 22 kHz, 150 W). The obtained lipid dispersions were mixed with 50 µL of 20 mg/mL *mcl*-PHA solution in *n*-hexane and sonicated in an argon stream for 5 min. The reactor scheme is shown in Figure 1. It consists of a sonicator probe and a reaction tube equipped with a needle for argon input. The side arm is used as the gas outlet. Both the probe and the reaction tube are submerged in a beaker with water.

The obtained PLN solutions were used for dynamic light scattering (DLS) analysis of the PLNs (Table 2) and to obtain polyplexes with the mRNA. As control samples, we used the CLs obtained from lipid mixtures without the addition of *mcl*-PHA under sonication for 5 min.

### 2.5. Preparation of mRNA Loaded PLNs and CLs

For transfection experiments, complexes of PLNs or CLs with mRNA were formed in MEM medium (Gibco Thermo Fisher Scientific, Waltham, MA, USA) without serum. To obtain the complexes of CLs containing 2X3 and DOPE with molar ratio 1:3 (2X3-DOPE 1:3) and mRNA, 5 µL of mRNA solution (0.1 μg) in DMEM and 5 μL of the CLs solution (molar concentration of 2X3 0.16 mM) in DMEM were mixed and incubated for 20 min at 25 °C [29]. The corresponding N/P ratio (molar ratio between positively charged cationic liposomes and negatively charged delivered mRNA molecules) was 10/1. Complexes of the PLNs with mRNA were prepared similarly by mixing of 5 µL of mRNA solution (0.1 μg) in DMEM and 5 μL of the PLNs dispersion followed by incubation for 20 min at 25 °C. To provide N/P ratios of 1/1, 2/1, 4/1, 6/1, 8/1, and 10/1. PLNs dispersions with molar concentrations of 2X3 0.016, 0.031, 0.066, 0.094, 0.13, and 0.16 mM were used.

Complexes of PLNs (PHA-2X3-DOPE 1:3) and mRNA at N/P ratios of 1/1, 2/1, 4/1, 6/1, 8/1, 10/1, 20/1, and 30/1 were studied by DLS. The solution of mRNA (0.1 μg) in nuclease-free water (25 µL) was mixed with 25 μL of PLNs in nuclease-free water, incubated for 20 min at 25 °C, and added to 950 μL sterile water. To provide the corresponding N/P ratios PLNs dispersions with molar concentrations of 2X3 3.1, 6.3, 13, 19, 25, 31, 63, and 94 μM were used.

### 2.6. Measurement of Particle Size and Zeta Potential

The hydrodynamic diameter, PDI, and ζ-Potential of CLs, PLNs, and polyplexes in sterile water were determined using a Malvern Nano ZS (Malvern Instruments, Malvern, UK). Polydispersity index (PDI) values less than 0.3 were taken as representing a monodisperse system. ζ-Potential was expressed in mV. All the measurements were repeated thrice.

### 2.7. Influence of Ionic Strength on the Stability of PLNs and CLs

To study the stability of the PLNs and CLs, their solutions (5 µL) were mixed with sterile water and a 2-fold PBS (300 mM). The final molar concentration of Na^+^ in the obtained mixtures was 0, 15, 30, 75, and 150 mM, and the total volume was 50 µL. The solutions were incubated for 2 h at 25 °C or 120 h at 4 °C. The particle size distribution was analyzed by the DLS in a 50 µL cuvette. To measure the ζ-potential, they were additionally diluted with PBS of the appropriate concentration to a volume of 750 µL.

### 2.8. Particle Morphology Analysis

An aliquot of PLNs or CLs (15 µL) was applied to a 300 mesh copper grid coated with collodion (Electron Microscopy Science, Hatfield, PA, USA) and incubated for 30 s; then, the sample was removed and the mesh was washed twice for 30 s with water, and then dried after a few seconds using filter paper. After removing the water, the sample was contrasted in a 1.5% aqueous solution of phosphotungstic acid sodium salt for 30 s. Visualization was performed using a JEM 1100 transmission electron microscope (JEOL, Akishima, Tokyo, Japan) at an accelerating voltage of 80 kV.

### 2.9. Measurement of mRNA Encapsulation Efficiency

To analyze the complexation of the mRNA with the PLNs, complexes were obtained with N/P ratios 1/1, 2/1, and 4/1 as described above. They contained 50 ng mRNA (sample volume 1 µL). The mRNA binding with PLNs was determined by capillary electrophoresis under non-denaturing conditions using the Agilent RNA 6000 Nano Kit (#5067-1511) and Agilent 2100 Bioanalyzer (Agilent Technologies, St. Clara, CA, USA) according to the manufacturer’s protocol. The results were analyzed with the 2100 Expert software version B.02.08.SI648 (SR2) (Agilent Technologies, St. Clara, CA, USA).

### 2.10. Transfection of Model mRNAs In Vitro

PLNs were tested for the efficiency of the intracellular delivery of a model mRNAs (mRNA-eGFP or mRNA-Cy5) to the BHK-21 adhesive cell line. BHK-21 cells were purchased from ATCC (Manassas, VA, USA). The cells were routinely cultured and expanded at 37 °C in MEM and supplemented with 4 mM L-glutamine, 1 mM sodium pyruvate, and 5% fetal bovine serum in an atmosphere of 5% CO_2_.

The day before transfection, the BHK-21 cells were cultured in 96-well plates under normal growth conditions (density 15 × 10^3^ cells per well). On the day of transfection complexes of the PLNs or CLs with the mRNA in a volume of 10 μL were added and cells were incubated for 24 h.

### 2.11. Fluorescence Microscopy

For analysis, the BHK-21 cells were transfected with the mRNA-Cy5 complexes as described above. At 24 h post-transfection, nuclei were stained with the NucBlue™ Live Ready-Probes™ Reagent (Hoechst 33342) (Invitrogen, Carlsbad, CA, USA) according to the manufacturer’s instructions. Then, the cells were washed in PBS to eliminate free fluorescent mRNA molecules and complexes outside the cells. The cells were immediately imaged with a 10× objective using a Cytell Cell Imaging System Cytell™ (GE Healthcare Life Sciences, Little Chalfont, UK).

### 2.12. Flow Cytometry Analysis

Delivery of mRNA-eGFP was performed as described above in 96-well plates. After incubation of cells for 24 h with polyplexes or lipoplexes, the cells were removed with 0.05% trypsin-EDTA (Gibco Thermo Fisher Scientific, Waltham, MA, USA) and washed with PBS solution. To detect the level of Cy5 or GFP expression, 10,000 events per sample were processed in separate cells and obtained using a CytoFLEX flow cytometer (Beckman Coulter Indianapolis, IN, USA). The results were analyzed in the Kaluza software (Beckman Coulter, Indianapolis, IN, USA) and were expressed as the mean and standard deviation obtained from three repeats.

### 2.13. Statistical Analysis

The confidence intervals for the DLS analysis results were calculated with the Student’s *t*-test (*p* < 0.05). The statistical analysis for flow cytometry was performed using one-way or two-way ANOVA tests.

## 3. Results

### 3.1. Preparation and Characterization of mcl-Polyhydroxyalkanoate

*mcl*-PHA polymer used in this study was obtained using *Pseudomonas helmanticensis* P1. Previously, we showed that this strain is an efficient producer of *mcl*-PHA [24,30]. The cultivation was carried out in a mineral medium with sodium oleate and stearate obtained by alkaline hydrolysis of vegetable oil serving as the carbon source. *P. helmanticensis* P1 dry biomass contained 21% *mcl*-PHA. The resulting *mcl*-PHA polymer was a copolymer consisting of 3-hydroxyhexanoate (3HH, 6%), 3-hydroxyoctanoate (3HO, 49%), 3-hydroxydecanoate (3HD, 26%), and 3-hydroxydodecanoate (3HDD, 24%). The polymer sample had weight-average molecular weight of 100 kDa, number-average molecular weight of 67 kDa, polydispersity index of 1.5, and purity of >98%.

### 3.2. Preparation and Analysis of the Hybrid PLNs Physicochemical Parameters

For our study, we prepared four types of PLNs, containing 2X3 (PHA-2X**3**) or 2X3 and DOPE with molar lipid ratios 1:1 (PHA-2X3-DOPE 1:1), 1:2 (PHA-2X3-DOPE 1:2), and 1:3 (PHA-2X3-DOPE 1:3).

The proposed method of PLNs synthesis is based on a modified oil-in-water emulsion method. The standard method consists of three steps: preparation of a polymer solution in an organic solvent, preparation of an emulsion of this solution in water in the presence of a stabilizer [31], and removal of the organic solvent from the emulsion [32]. During the preparation of the dispersion, droplets of the polymer solution are dispersed in the aqueous phase by stirring or sonication. The solvent is then evaporated, turning the emulsion droplets into polymer particles. In the proposed method, the organic solvent is removed in the argon stream directly when the polymer solution is dispersed in the aqueous phase under sonication. Thus, it is possible to reduce the number of NP synthesis stages and avoid the possibility of their contamination with nucleases and bacterial agents during evaporation on a rotary evaporator.

Preparation of the PLNs composed of *mcl*-PHA and Tween 80 [31] showed that the optimal ratio between *mcl*-PHA and surfactant is 20:1 (wt.). Similar results have been obtained for polycationic lipid 2X3 or its mixtures with the helper lipid DOPE (Table 2). A ratio of 20:1 (wt.) leads to the formation of PLNs with the smallest diameter. This ensures not only their stability, but also contributes to the effective uptake of the PLNs by the cell. So, PLNs with a PHA/lipids ratio of 20:1 (wt.) were used in all further experiments. It was shown that the ζ-potential depends to a small extent on the PHA/lipids mass ratio. It ranged from 42 to 59 mV for all the PLN samples.

It should be mentioned that the DOPE content is essential for the formation of monodisperse PLNs. 2X3 and *mcl*-PHA without DOPE form NPs with PDI > 0.3 at all PHA/lipids ratios. Such systems cannot be considered as monodisperse and usually they do not reveal the aggregative stability [27].

### 3.3. Preparation of mRNA Complexes with PLNs

**Figure 2:**
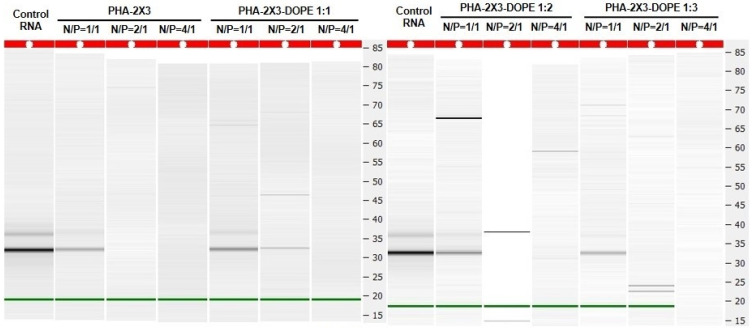
Analysis of mRNA binding with PLNs by capillary electrophoresis. Control RNA—control mRNA samples without PLNs containing 50 ng of mRNA. PHA-2X3—complexes of mRNA with PLNs stabilized by 2X3; PHA-2X3-DOPE 1:1—complexes of mRNA with PLNs stabilized by a mixture of 2X3 and DOPE in a molar ratio of 1:1; PHA-2X3-DOPE 1:2—complexes of mRNA with PLNs stabilized with a mixture of 2X3 and DOPE in a molar ratio of 1:2. PHA-2X3-DOPE 1:3—complexes of mRNA with PLNs stabilized with a mixture of 2X3 and DOPE in a molar ratio of 1:3. PHA/lipids ratio in all the PLNs was 20:1 (wt.). Green line—signal of the fluorescent dye (used as an internal control for the electrophoresis). N/P — molar ratio between positively charged cationic liposomes and negatively charged delivered mRNA molecules.

Efficient binding of the PLNs to the model mRNA-eGFP (Figure 2) occurs at an N/P ratio of ≥2/1. By comparing the previous data of the binding of CLs with mRNA-eGFP [29], we found that the presence of *mcl*-PHA promotes the binding with mRNA at lower N/P ratios. It can be supposed that the binding enhancement is due to increased electrostatic interaction between mRNA and polycationic lipid 2X3 in the presence of a PHA core.

### 3.4. Effect of the Hybrid PLNs’ Compositions on the Efficiency of mRNA Delivery into Eukaryotic Cells

The obtained PLNs and CLs were studied for the delivery of a model mRNA encoding the green fluorescent protein (eGFP) into BHK-21 cells. The mRNA-eGFP construct was chosen as a qualitative model to assess both the efficiency of intracellular delivery and the expression of the target protein.

**Figure 3:**
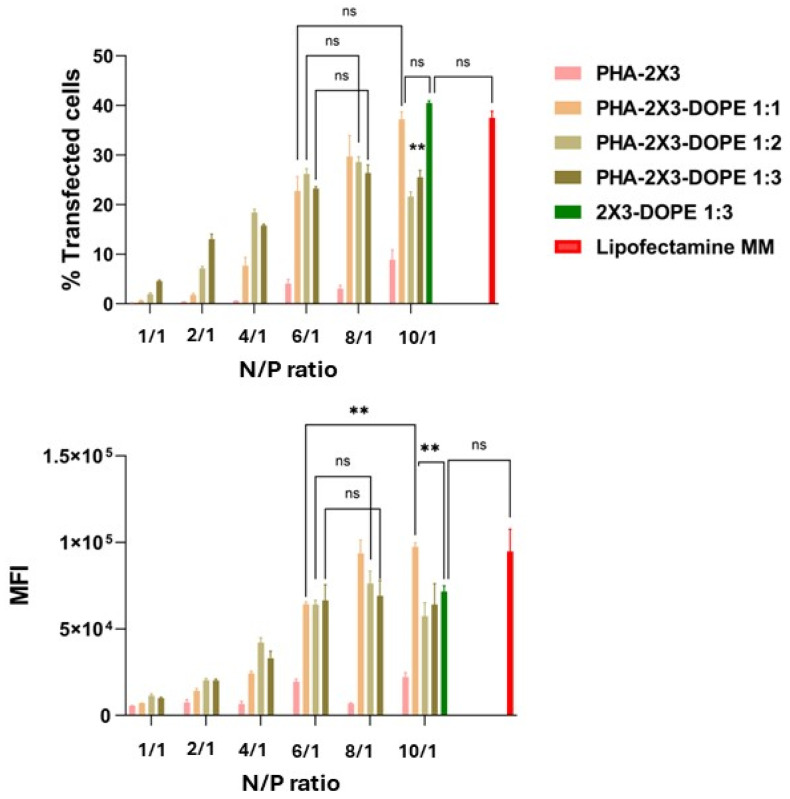
The efficiency of mRNA-eGFP delivery using CLs and PLNs in BHK-21 cells was measured using flow cytometry. The transfected cells were analyzed for the percentage of cells with detectable eGFP signals, and the MFI (mean fluorescence intensity) was recorded. Lipofectamine MessengerMAX (Lipofectamine MM) is a commercial transfection reagent and was used as a positive control for mRNA transfection. All the measurements were triplicated. The statistical analysis was performed using a two-way ANOVA: **—*p* < 0.01; not significant—‘ns’.

The efficiency of intracellular delivery of NAs to eukaryotic cells is dependent not only on the composition of the carriers and their physicochemical properties, but also on the N/P ratio (molar ratio between positively charged cationic liposomes and negatively charged delivered mRNA molecules). We have shown that starting from N/P 2/1, all mRNA complexes with PLNs completely encapsulated the mRNA (Figure 2). Based on the data obtained, we have selected N/P ratios of 1/1, 2/1, 4/1, 6/1, 8/1 and 10/1 to identify the most efficient N/P. Complexes of CLs 2X3-DOPE 1:3 and mRNA-eGFP at N/P 10/1, demonstrated to be the most efficient at mRNA delivery in our previous study [29], as well as the commercial transfection reagent Lipofectamine MessengerMAX were used as positive controls. It was shown that the transfection efficiency largely depends on the N/P ratio (Figure 3). At lower ratios (1/1, 2/1, and 4/1) the percentage of transfected cells was less than 20%, and the MFI was two times lower than in the positive control (Lipofectamine MM). N/P ratios of 6/1 and higher provide mRNA-eGFP transfection efficiency comparable (PHA-2X3-DOPE 1:1) or slightly lower (PHA-2X3-DOPE 1:2 and PHA-2X3-DOPE 1:3) than CLs and the commercial transfection reagent (Figure 3).

The presence of DOPE turned out to be essential for efficient mRNA-eGFP delivery and translation. PLNs that contained only polycationic lipid 2X3 and PHA did not ensure a sufficient percentage of transfected cells (<10% at all N/P) and had low MFI values (four times less than Lipofectamine MM). As mentioned above, such PLNs without a helper lipid do not reveal aggregative stability (PDI = 0.4, Table 2). It might be assumed that they also do not form the stable polyplexes required for efficient mRNA delivery.

Previously, we found that CLs 2X3-DOPE 1:3, when compared to 2X3-DOPE 1:1 and 2X3-DOPE 1:2, ensured the best mRNA delivery. We also showed that increasing the proportion of helper lipid DOPE in the CL’s composition leads to an increase in the transfection efficiency of the mRNA [27]. Interestingly, PLNs containing DOPE have almost an equal delivery efficiency at N/P 6/1 and 8/1 without respect to 2X3/DOPE molar ratio. Only at N/P 10/1 PLNs with PHA-2X3-DOPE 1:1 revealed better mRNA delivery and translation. As it was mentioned, CLs with 2X3-DOPE 1:3 and mRNA-eGFP at N/P 10/1 demonstrated the most efficient mRNA delivery in our previous study. Their PLN analogue (PHA-2X3-DOPE 1:3) demonstrated good mRNA delivery without respect to slight N/P changes. At N/P 6/1, 8/1, and 10/1 the percentage of transfected cells and the MFI are almost the same. Such low sensitivity to N/P allows obtaining more stable results, which is essential for further applications of PLNs. So, it was decided to study PHA-2X3-DOPE 1:3 polyplexes for physicochemical properties and the uptake of mRNA-Cy5.

**Figure 4:**
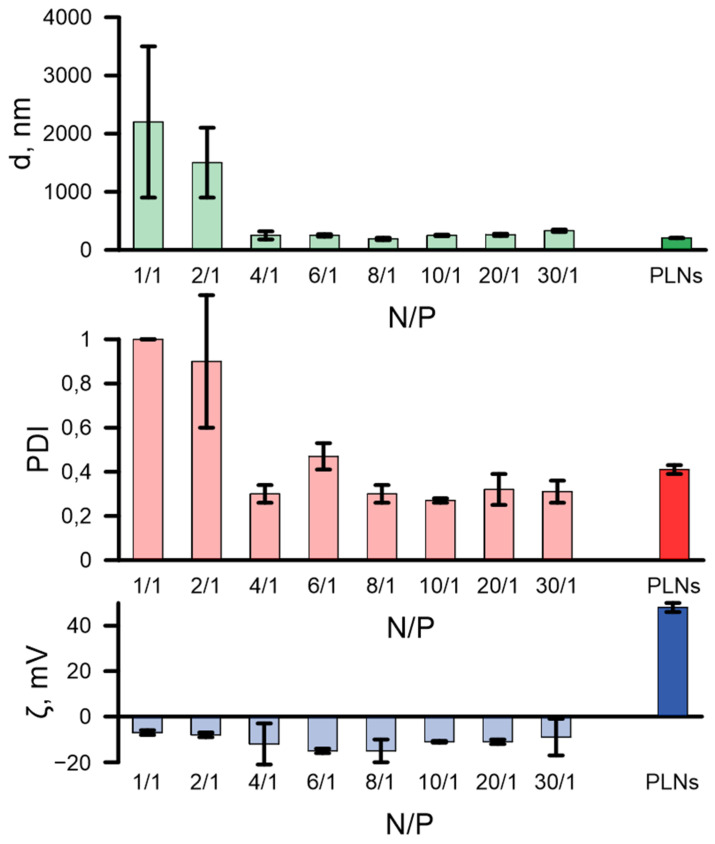
Dependences of the average particle diameter (d, nm), polydispersity (PDI), and ζ-potential (ζ, mV) on the N/P. Complexes of the PLNs (PHA-2X3-DOPE 1:3) and mRNA-eGFP were analyzed. PLNs (PHA-2X3-DOPE 1:3) were used as a control.

DLS analysis of the polyplexes with PHA-2X3-DOPE 1:3 revealed that at low N/P (1/1 and 2/1) aggregation takes place (Figure 4). Such aggregation might be due to the neutralization of positive charges in 2X3 by the mRNA phosphate groups. At N/P 8/1 and 10/1, the lowest hydrodynamic diameters and PDIs were observed. It is interesting to note that at these N/P the best mRNA delivery occurs. Negative ζ-potential at all N/P (even 20/1 and 30/1) makes us assume a difference in complexation mechanism compared to CLs. Usually lipoplexes with high N/P (e.g., 30/1) are positively charged, since the negative mRNA phosphates are shielded by lipid bilayer [29]. In the case of the PLNs, this shielding does not take place and another complexation mechanism might be supposed.

**Figure 5:**
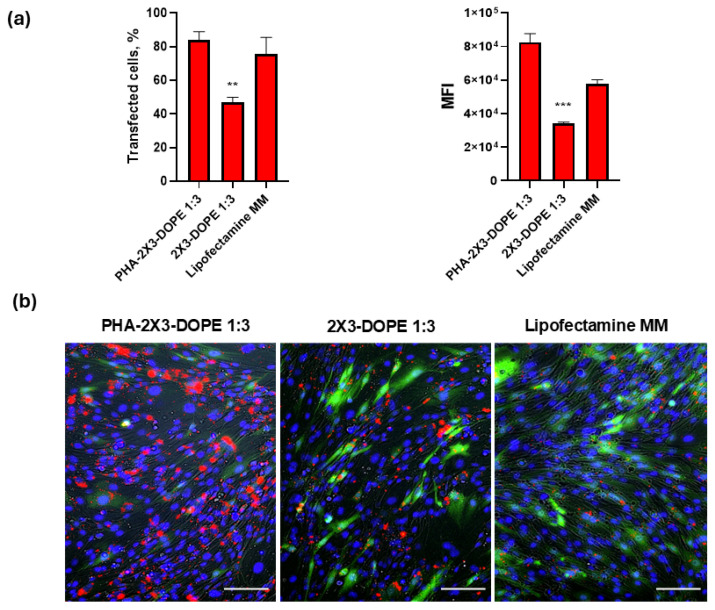
(**a**) Efficiency of mRNA-Cy5 uptake with PLNs and CLs by BHK-21 cells determined by flow cytometry. Transfected cells—the percentage of fluorescent cells (Cy5 signal); MFI—mean fluorescence intensity. Statistical analysis was performed using one-way ANOVA: ***—*p* < 0.001; **—*p* < 0.01. (**b**) Fluorescence microscopy of cells transfected with mRNA-Cy5 mRNA complexes using PHA-2X3-DOPE 1:3, 2X3-DOPE 1:3, and Lipofectamine Messenger MAX (Lipofectamine MM); Scale bar 50 μm; cell nuclei–blue, eGFP—green, and mRNA–Cy5—red.

Complexes of the mRNA with the PLNs and CLs were also tested for their uptake by the BHK-21 cells with the model mRNA-Cy5. The highest uptake efficiency for mRNA-Cy5 was achieved by PHA-2X3-DOPE 1:3 (80.2 ± 0.7%). The percentage of transfected cells is almost equal for PLNs and Lipofectamine MM (Figure 5a). However, the MFI of Cy5-labeled mRNA was thrice higher in the case of PLNs compared to CLs and the commercial transfection reagent (Figure 5a). Fluorescence microscopy confirmed better mRNA-Cy5 uptake in the case of PLNs; a large difference in fluorescence intensity between polyplexes and positive controls was observed (Figure 5b).

Thus, the resulting PLNs demonstrated efficient delivery of the model mRNAs (mRNA-eGFP and mRNA-Cy5) to the BHK-21 cells. The presence of the helper lipid DOPE in the PLNs is crucial for effective mRNA delivery and translation. However, different molar ratio, 2X3 and DOPE does not affect transfection efficiency as, how it was observed in the previous study of CLs [29]. Unlike CLs, which delivered mRNA most efficiently at N/P = 10/1, PLNs PHA-2X3-DOPE delivered mRNA-eGFP equally efficiently at a ratio 6/1 or higher. Considering the observed difference in the optimal N/P ratio for mRNA-eGFP delivery between the CLs and the PLNs, as well as the more efficient uptake of the fluorescently labeled mRNA polyplex, it can be assumed that there is a change in the complexation mechanism between the carrier and mRNA that takes place when the hydrophobic *mcl*-PHA core is introduced.

It is interesting that the inversion of the ζ-potential of the polyplexes (see Figure 4) leads to an increase in the uptake efficiency, whereas it does not affect the transfection efficiency.

### 3.5. mcl-PHA Ensures the Stability of the Hybrid PLNs in an Ionic Solution

We performed a comparative study of an ionic solution’s (PBS) effect on the stability of two types of cationic carriers: PLNs (PHA-2X3-DOPE 1:3) and CLs (2X3-DOPE 1:3). The choice was made because we have previously shown the CLs 2X3-DOPE (1:3) provide more efficient delivery of mRNA-eGFP into the BHK-21 cells compared to 2X3-DOPE (1:1) and 2X3-DOPE (1:2) [29].

**Figure 6:**
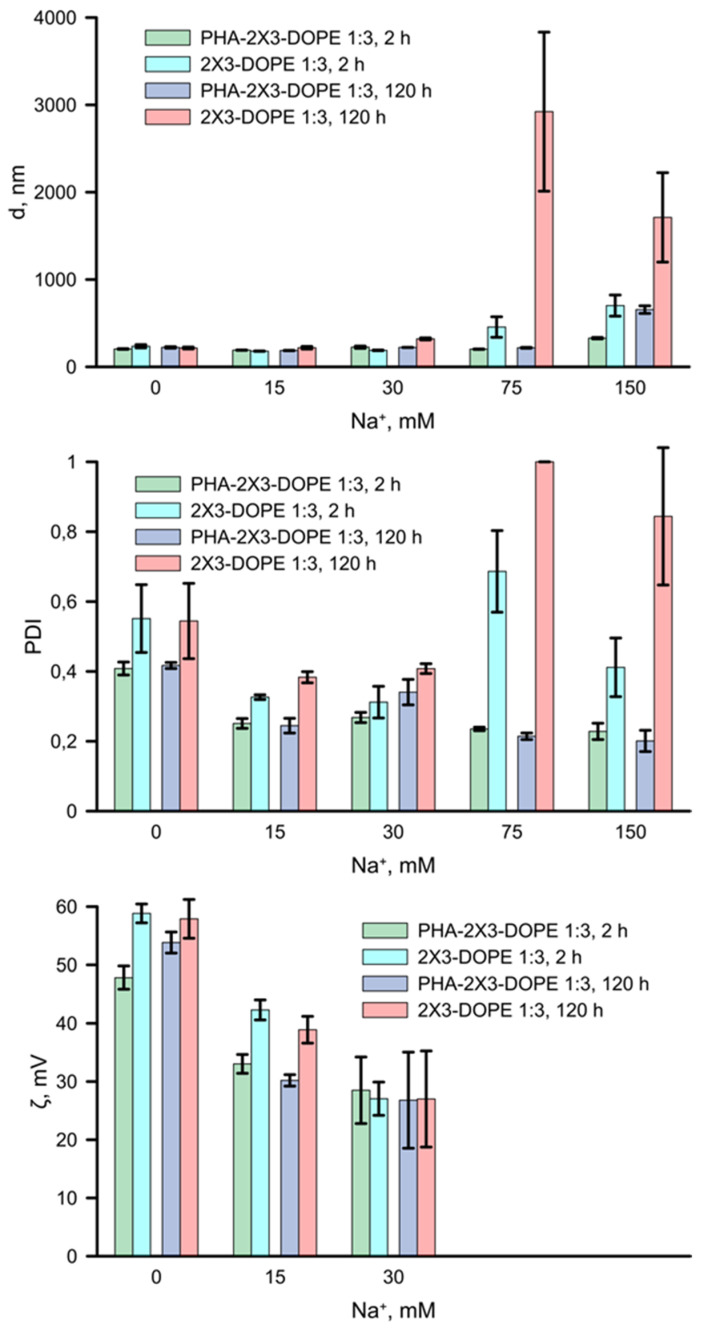
Dependences of the average particle diameter (d, nm), polydispersity (PDI) and ζ-potential (ζ, mV) on the molar concentration of Na^+^ in PBS. PLNs (PHA-2X3-DOPE 1:3) or CLs (2X3-DOPE 1:3) were mixed with PBS and incubated for either 2 h at 25 °C or 5 days at 4 °C.

The role of *mcl*-PHA in increasing the stability of cationic carriers in PBS was evaluated by DLS (Figure 6). The decrease in particle size and PDI at Na^+^ concentrations of 30 mM for CLs (236 nm and 0.55 in sterile water and 189 nm and 0.31 in 30 mM, respectively; 2 h of incubation) can probably be explained by osmotic compression, due to the elastic nature of the membranes. As the Na^+^ concentration increases, the size and PDI values of CLs increase, and at concentrations of 75 mM and 150 mM, they aggregate (456 nm and 0.69 in 75 mM, 702 nm and 0.41 in 150 mM, respectively; 2 h of incubation). CLs’ storage at 4 °C in 75 mM and 150 mM solution leads to their further aggregation; hydrodynamic diameter increases of 6.4 (75 mM) and 2.4 (150 mM) times occurred. PDI also rises up to 1.0. These results confirm the weak stability of CLs in solutions with high ionic strength. At the same time, the PDI value for particles containing *mcl*-PHA decreases in diluted PBS (0.41 in sterile water and 0.27 in 30 mM; 2 h of incubation) and the particles are stable in 75 mM solution for 120 h (218 nm and PDI 0.21 after 5 days of storage). Short incubation of PLNs in isotonic (150 mM) PBS does not result in significant aggregation (328 nm and PDI 0.23). However, 5 days storage at 4 °C in isotonic PBS causes enlargement of the PLNs to 655 nm with a PDI 0.20.

We evaluated the effect of the PBS concentration on the ζ-potential of CLs and PLNs (Figure 6). The results showed a consistent decrease in the ζ-potential as the buffer concentration rose, which is completely confirmed by the Gouy–Chapman theory [33]. Electrostatic repulsion is sensitive to the electrolyte concentration, while steric repulsion is sensitive to changes in the solubility and molar mass of the adsorbed polymer, such as PEG [34]. Published data show that the ζ-potential of lipid carriers declines with increasing electrolyte concentration in the medium [35,36]. The liposome radius falls down with rising ionic strength, leading to electrostatic shielding and a decrease in the observed electrostatic potential [37]. In our experiment, we also observed a decline in the ζ-potential, as shown in Figure 6.

**Figure 7:**
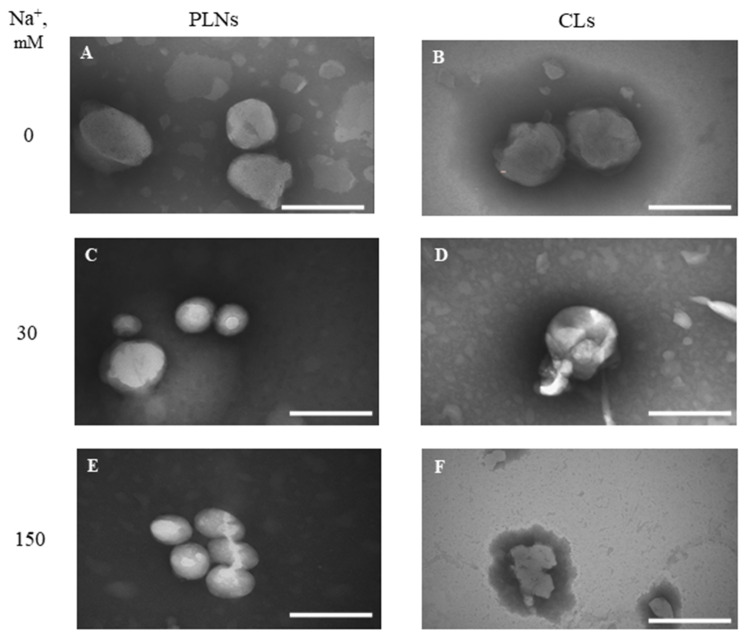
Transmission electron microscopy of PLNs (PHA-2X3-DOPE 1:3) and CLs (2X3-DOPE 1:3) at various Na^+^ concentrations (0, 30, and 150 mM; incubation time 2 h). Scale bar: 150 nm (**A,B**), 100 nm (**C,E**), 200 nm (**D**), 500 nm (**F**).

Changes in the morphology of the PLNs and CLs in PBS were studied by transmission electron microscopy (TEM) (Figure 7). In the obtained images, multi-lamellar structures are observed around the PLNs in an aqueous solution without PBS (Figure 7A). It is difficult to say for sure whether they are the result of the construction of onion-like lipid structures [38] or whether they are formed during sample preparation. At the same time, the PLNs’ microscopy in ionic solutions shows an almost complete absence of such structures, and on the surface of the *mcl*-PHA particles, as a rule, low-lamellar structures (monolayer or bilayer) are formed (Figure 7E). It is important to note that with an increase in the PBS concentration the PLNs form well-defined spherical structures containing the hydrophobic *mcl*-PHA core. At the same time, as Na^+^ concentration increases, CLs are strongly deformed (Figure 7D) and at 150 mM Na^+^ they aggregate to multilayer structures with an approximate diameter of 500 nm (Figure 7F). The data from the DLS and TEM analyses are in a good agreement, and the stabilizing role of *mcl*-PHA in cationic PLNs was successfully shown. However, the reason for the formation of multilamellar structures during TEM visualization is unclear, and this phenomenon deserves further investigation.

## 4. Discussion

There are almost no studies on intracellular mRNA delivery using PLNs prepared from natural, uncharged hydrophobic polymers and CLs. The synthesis of these carriers is a promising area for the development of biodegradable and biocompatible delivery systems. In our previous study [29], we showed that CLs 2X3-DOPE (1:3) were effective carriers for mRNA encoding eGFP and FLuc. Additionally, we previously demonstrated the cell uptake efficiency of Tween 80-stabilized *mcl*-PHA nanoparticles [31]. In this study, we showed that PLNs prepared using 2X3-DOPE cationic liposomes and the hydrophobic polymer *mcl*-PHA isolated from the soil bacterium *Pseudomonas helmantisensis* P1 [24] effectively encapsulate and deliver the model mRNA-eGFP into BHK-21 cells (Figure 3 and Figure 5). It was demonstrated that the PLNs complexed with mRNA and used for delivery are not toxic for the BHK-21 cell line.

Surface charges play a key role in the formation of a lipid layer on the PLNs. It is well known that lipoplexes with positive charges provide higher transfection efficiency since they interact more effectively with a negatively charged cell surface [39]. Additionally, the charge ratio CLs/mRNA (N/P) is a key factor that affects the efficiency of NA delivery [40]. It is interesting to note that in the formation of the mRNA complexes with the PLNs that there is a significant decrease in the surface charge of the polyplexes; negative ζ-potential does not depend on the N/P ratio (Figure 4) and remains negative even with a 30-fold molar excess of positively charged nitrogen atoms to negatively charged phosphate groups (Figure 3). At the same time, the efficiency of the mRNA encapsulation in the polyplex does not change and remains high (Figure 2) and the PLNs exhibit more efficient uptake of fluorescently labeled mRNA-Cy5 (Figure 5a) and effective delivery of mRNA-eGFP to the BHK-21 cells (Figure 3). A similar inversion of the ζ-potential of mRNA polyplexes to negative values was observed when DSPC/DOTAP lipids were used as the CL and polylactide was used as a hydrophobic core [22]. However, the authors have shown that the efficiency of mRNA delivery by these polymer–lipid carriers to dendritic DC 2.4 cells and HeLa epithelial cells is three orders of magnitude lower than in the control experiment using Lipofectamine 2000. In our study, the effective mRNA transfection using PLNs may be explained by the stable hydrophobic interaction between the laminar structures formed by 2X3 and DOPE lipids and the hydrophobic polymer *mcl*-PHA under neutral pH. Lipid nanoparticles enter the cell primarily through endocytosis [41]. As endosomes mature, there is a decrease in pH. At physiological pH, lipid-based NPs containing DOPE maintain a stable lamellar phase that provides efficient encapsulation of therapeutic agents [42]. The hydration of the DOPE ethanolamine headgroup is weaker compared to the other phospholipids as it is more hydrophobic and has a lower diameter. Thus, membranes containing DOPE demonstrate less overall hydration, tighter lipid packing, and decreased permeability for water. Under acidic conditions, during endosome maturation in cells, DOPE forms an inverted hexagonal phase (HII) that leads to the loss of the liposomes’ spherical structure [43] and induces the release of encapsulated molecules. This phase transition is a key element in triggering the endosomal destabilization that provides cytoplasmic delivery of the drug payload [44]. We can suggest that in case of previously studied polyplexes, the hydrophobic interaction between 2X3, DOPE lipids, and the *mcl*-PHA is destabilized in the intracellular environment when DOPE interacts with the endosomal membrane, leading to the release of mRNA. Moreover, a weak negative charge on the polyplex surface (Figure 4) can increase the delivery efficiency in vivo, which requires further investigation. It can be assumed that the presence of a negative ζ-potential would increase the stability of the obtained complexes during in vivo studies and thus eliminate the need for PEGylated PLNs.

In our previous study we demonstrated that mRNA transfection efficiency mediated by CLs (2X3-DOPE) depends on both the 2X3:DOPE molar ratio and N/P ratio. As the molar proportion of DOPE in CLs based on 2X3 increases, the efficiency of mRNA transfection also increases [29]. Our experiments have shown that the presence of DOPE in the PLN significantly affects the transfection efficiency by PLNs. However, surprisingly, the molar ratio of 2X3 to DOPE does not seem to affect the transfection efficiency at different N/P ratios, as shown in Figure 3. In another study, it was found that an increase of DOPE content in a cationic lipid layer with DOTMA surrounding the PLGA core facilitates uptake and mRNA transfection by PLNs into dendritic cells [2]. PHA is believed to be more hydrophobic compared to PLGA. The higher hydrophobicity of PHA may lead to the enhancement of hydrophobic interactions and stabilization of the lipid structures on the surface of the NP polymer. In such a case, the effect of DOPE on the stability of the polyplexes within the cell’s endosomes might decrease, and the other factors may play the key role in the mRNA release. The mechanisms of mRNA release from polyplexes and the key factors that influence the polyplex stability in endosomes are worth further investigation.

The mechanisms of interaction between lipids and polymer NPs during the formation of hybrid PLNs are not clearly described. Lipid molecules spontaneously assemble on the surface of the polymer particles due to hydrophobic interactions, forming a monolayer [19] or several lipid layers (multilamellar structures). In our case, the PLNs obtained in an aqueous solution are mainly coated with multilamellar structures formed by polycationic 2X3 lipid and the helper lipid DOPE (Figure 7A). The presence of such structures increases the size of the PLNs and the polydispersity. At the same time, the addition of PBS to PLNs solution induces the enhancement of the hydrophobic interaction between the *mcl*-PHA surface and the hydrocarbon chain of the lipids. It leads to a change in the multilayer structure and to the formation of PLNs covered with a lipid monolayer (Figure 7C,E). During the reorganization of multilamellar structures on the PLN surface, the hydrodynamic size and PDI decrease.

Today, there are not any published studies on the effect of *mcl*-PHA on the stability of hybrid PLNs in different ionic solutions. We carried out the comparative study of the effect of ionic strength on the size and PDI of 2X3-DOPE (1:3) and PHA-2X3-DOPE (1:3) (Figure 6). The investigation of the physical parameters of the particles revealed inverse correlations for CLs and PLNs at high PBS concentrations. At 15 mM and 30 mM Na^+^ concentrations, a decrease in particle size was observed for both types of particles. This can probably be explained by the osmotic pressure, due to the elastic nature of the membranes [36]. As the Na^+^ concentration increased, size and PDI values of CLs rose, and at 75 mM and 150 mM Na^+^ they aggregated. It may be explained by a decrease in the solubility of phospholipids and the shielding of cationic phospholipid head groups [37]. A decrease in the solubility of phospholipids occurs due to the salting effect, since water reorientation occurs when salt is added [37]. With an increase in the ionic strength, water molecules rearrange, solvating the added salt ions and causing the dissolution of the hydrate shell [45]. This phenomenon of water migration reduces the solubility of phospholipids, increasing the hydrophobic effect [46]), which leads to the aggregation of the CLs (Figure 7F). It was previously shown that in a PBS solution, the amino groups of lipids in CLs are shielded by anions, which reduce the electrostatic repulsion between them and contribute to Van der Waals interactions that lead to aggregation [36]. In such a case, a significant increase in the CLs’ polydispersity occurs; PDI becomes >0.6, which confirms, together with the TEM data, the described mechanism of the NPs’ aggregation at high ionic strength. The size of the PLNs containing *mcl*-PHA decreases with rising ionic strength. At the same time, the particles are stable for 120 h even in 150 mM Na^+^. Taking into account the described influence of the ionic strength on hydrophobicity and the experimental data, the presence of the hydrophobic *mcl*-PHA core stabilizes the PLNs and prevents Van der Waals interactions leading to particle aggregation.

It is interesting to note the effect of ionic strength on the PDI index (Figure 6). A higher concentration of PBS in the PLNs solution led to a decrease in the PDI (below 0.3) compared to ultrapure water. After 120 h of storage, the PLNs remained monodisperse at sodium concentrations of 75 mM and 150 mM (PDI~0.2). In contrast, CLs at these salt concentrations had PDI above 0.4 after 2 h of incubation and had PDI above 0.8 after 120 h at +4 °C. This result may also be explained by the effect of Na^+^ concentration on the increase in the role of hydrophobic interactions. The effects of solubility reduction and hindrance of cationic amino groups act synergistically, resulting in fall of ζ-potential and collision of the CLs. As a result, we observe the CLs’ aggregation (Figure 7F). Probably, at a high PBS concentration, phospholipid–phospholipid interactions in cationic liposomes are regulated by Van der Waals forces, and since the repulsive forces are significantly reduced due to ion hindrance, the aggregation occurs. Where in the case of PLNs, the opposite situation is observed. There is a rearrangement of the NPs and a decrease in multilamillarity on the *mcl*-PHA particle surface takes place (Figure 7C,E). The interpretation of our results is supported by the experimental and theoretical data [36,38].

In addition, electrostatic and steric stabilization are two fundamental mechanisms responsible for the physical stability of lipid NPs [47,48]. Electrostatic stabilization depends on the ζ-potential, which is proportional to it, and is based on the mutual repulsion between liposomes [49]. According to our data, as the ionic strength of the solution rises, the ζ-potential of both types of particles decreases, which may lead to a decrease in the colloidal stability of the NPs (Figure 7F). However, the physical properties of the PLNs remain stable even when they are stored in a 75 mM Na^+^ solution at 4 °C (Figure 6). This result confirms the previously described effect of PLNs’ stabilization, which occurs due to the increased affinity of the cationic lipid to the anionic surface of the hydrophobic particle [38]. As the ionic strength increases, the interaction between the 2X3 cationic lipid and the anionic carboxyl groups on *mcl*-PHA also enhances, leading to a reduction in the number of lipid layers surrounding the polymer particle and thus stabilizing the NPs. Conversely, for CL, an increase in ionic strength results in a weakening of electrostatic interactions, which can lead to the NPs’ aggregation. Our results show that *mcl*-PHAs play an important role in increasing the stability of cationic polymer–lipid carriers in ionic solutions. These results expand our understanding of the synthesis and application of hybrid cationic PLNs for intracellular delivery of NAs.

## 5. Conclusions

In this study, we have demonstrated for the first time the potential of the biodegradable and biocompatible natural polymer *mcl*-PHA to form cationic polymer–lipid nanoparticles for intracellular mRNA delivery. We have shown that a relatively simple technique allows synthesizing non-toxic PLNs based on cationic liposomes (2X3-DOPE (1:3)) and *mcl*-PHA. These PLNs efficiently deliver the model mRNAs to eukaryotic cells and ensure the expression of the target protein. Additionally, we demonstrated the stability of the PLNs in high ionic strength solutions. These stable PLNs could be promising carriers for mRNA delivery in vivo without the need for additional PEGylation.

## Figures and Tables

**Table 1:** Volumes of stock solutions used in nanoparticle synthesis; 2X3—volume of 2X3 solution (1 mg/mL in CHCl_3_/MeOH 4:1); DOPE—volume of DOPE solution (1 mg/mL in CHCl_3_/MeOH 4:1).

2X3/DOPE Mol. Ratio	2X3, µL	DOPE, µL
1:0	50	-
1:1	32.4	17.6
1:2	24.0	26.0
1:3	19.1	30.9

**Table 2:** Dynamic light scattering analysis results for PLNs and CLs. d—particle diameter (nm); ζ—electrokinetic potential (mV); PDI—polydispersity index.

2X3/DOPE, Molar Ratio	PHA/Lipids, Mass Ratio	d, nm	PDI	ζ, mV
1:0	0:1	430 ± 30	0.75 ± 0.04	38 ± 1
	10:1	286 ± 35	0.63 ± 0.06	53 ± 3
	20:1	237 ± 9	0.45 ± 0.10	51 ± 4
	50:1	1198 ± 433	0.55 ± 0.06	46 ± 4
1:1	0:1	234 ± 6	0.40 ± 0.01	56 ± 2
	10:1	227 ± 4	0.36 ± 0.01	57 ± 2
	20:1	194 ± 4	0.24 ± 0.03	52 ± 3
	50:1	228 ± 8	0.22 ± 0.06	56 ± 1
1:2	0:1	201 ± 4	0.54 ± 0.12	42 ± 1
	10:1	271 ± 10	0.41 ± 0.04	56 ± 2
	20:1	201 ± 2	0.26 ± 0.03	47 ± 1
	50:1	231 ± 2	0.26 ± 0.03	42 ± 4
1:3	0:1	236 ± 20	0.55 ± 0.10	59 ± 2
	10:1	290 ± 16	0.46 ± 0.01	59 ± 3
	20:1	205 ± 4	0.41 ± 0.02	48 ± 2
	50:1	207 ± 10	0.29 ± 0.07	54 ± 2

## Data Availability

The data that support the findings of this study are available from the corresponding author, S.M.S., upon reasonable request.
